# AnnoGCD: a generalized category discovery framework for automatic cell type annotation

**DOI:** 10.1093/nargab/lqae166

**Published:** 2024-12-04

**Authors:** Francesco Ceccarelli, Pietro Liò, Sean B Holden

**Affiliations:** Department of Computer Science and Technology, University of Cambridge, 15 JJ Thomson Ave, CB3 0FD, Cambridge, UK; Department of Computer Science and Technology, University of Cambridge, 15 JJ Thomson Ave, CB3 0FD, Cambridge, UK; Department of Computer Science and Technology, University of Cambridge, 15 JJ Thomson Ave, CB3 0FD, Cambridge, UK

## Abstract

The identification of cell types in single-cell RNA sequencing (scRNA-seq) data is a critical task in understanding complex biological systems. Traditional supervised machine learning methods rely on large, well-labeled datasets, which are often impractical to obtain in open-world scenarios due to budget constraints and incomplete information. To address these challenges, we propose a novel computational framework, named AnnoGCD, building on Generalized Category Discovery (GCD) and Anomaly Detection (AD) for automatic cell type annotation. Our semi-supervised method combines labeled and unlabeled data to accurately classify known cell types and to discover novel ones, even in imbalanced datasets. AnnoGCD includes a semi-supervised block to first classify known cell types, followed by an unsupervised block aimed at identifying and clustering novel cell types. We evaluated our approach on five human scRNA-seq datasets and a mouse model atlas, demonstrating superior performance in both known and novel cell type identification compared to existing methods. Our model also exhibited robustness in datasets with significant class imbalance. The results suggest that AnnoGCD is a powerful tool for the automatic annotation of cell types in scRNA-seq data, providing a scalable solution for biological research and clinical applications. Our code and the datasets used for evaluations are publicly available on GitHub: https://github.com/cecca46/AnnoGCD/.

## Introduction

**Figure 1.**
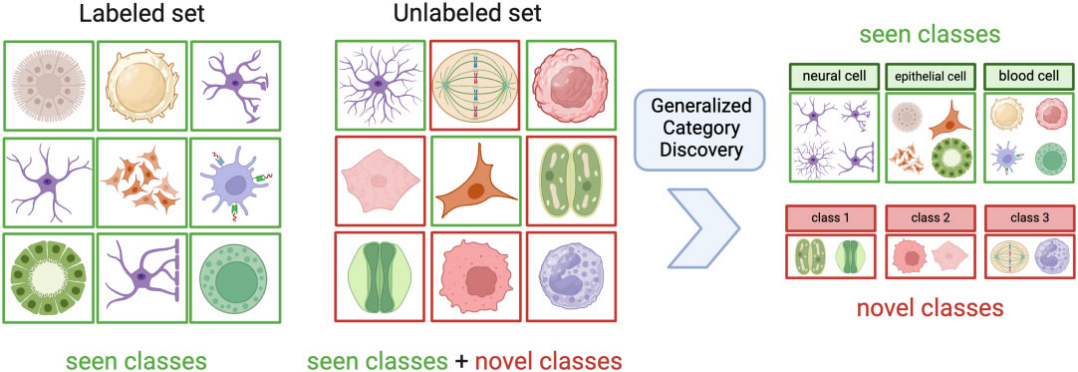
Generalized Category Discovery aims to correctly cluster samples in novel and known classes (for example neuronal cells) by utilizing knowledge from the labeled data.

When applied to the open-world, machine learning methods inevitably encounter both known and novel classes (1). Supervised approaches have received widespread attention and they rapidly progressed through the collection of large labeled datasets (2). These approaches normally assume that (i) the class distributions remain unchanged during the training and testing phase, and that (ii) all classes are known in advance and have some labeled examples. Both these assumptions are unrealistic for open-world contexts, where the labelling or collection of large numbers of samples might be difficult due to factors such as budget constraints or lack of comprehensive information. Novel Class Discovery (NCD) aims to identify clusters of unlabeled instances using a similar but disjoint set of labeled samples, as proposed in several works (3,4). However, NCD assumes that all the unlabeled samples belong to novel classes during the testing phase, thus disallowing the re-discovery of known classes in open-world settings. More recently, as shown in Figure 1, Generalized Category Discovery (GCD) extended NCD to further recognize the known classes in the unlabeled set (5). Unfortunately, most of the GCD community has focused almost exclusively on computer vision problems, leaving open the question of how to apply these frameworks to tabular data where it is not possible to take advantage of powerful computer vision techniques such as convolutions, Vision Transformers (6) or image augmentations. Furthermore, the easy access to a large body of image data (for example ImageNet (2), CIFAR-10 and CIFAR-100 (7)) allows most GCD frameworks to deal with well-balanced datasets, and to fully exploit the power of transfer learning. Unfortunately, imbalanced datasets are particularly common in real world scenarios, especially when dealing with biological data.

Along with GCD, Anomaly Detection (AD) has numerous real-world applications, including fraud detection, network security, and health monitoring, where detecting anomalous behavior is crucial for ensuring system integrity and reliability (8). AD methods encompass a wide range of techniques, such as statistical approaches, clustering algorithms, and deep learning models, each tailored to different data characteristics and application scenarios (9). An important setting for AD is when only labeled normal samples exist, for which one class classifiers (OCCs) (for instance Support Vector Machines (SVMs), Isolation Forests or autoencoders) are popular approaches. Compared to traditional binary or multi-class classification, OCCs are useful for addressing issues related to severely imbalanced datasets (10).

Cell type identification with statistical and machine learning methods has emerged as a promising approach in deciphering complex biological systems. Exploiting high-dimensional single-cell data, such as single-cell RNA sequencing (scRNA-seq), supervised machine learning algorithms have been used to classify cell types based on their transcriptional profiles (11). Various methods, from logistic regression to Convolutional Neural Networks (CNNs), have been successfully applied for cell type classification tasks (12). These techniques enable the extraction of intricate patterns from scRNA-seq data, facilitating the discrimination of different cell types with high accuracy. Despite their promising results, popular computational pipelines like SingleR (13) are either based on reference transcriptomic datasets or require fully supervised training (14). Consequently, their performance is highly dependent on the prior information selected or on the quality of the manually annotated labels.

Motivated by common real-world scenarios and by the need to improve annotation of cell types in scRNA-seq, we developed a computational method, named AnnoGCD, building on ideas from Generalized Category Discovery and Anomaly Detection. We propose a simple, albeit efficient, semi-supervised computational pipeline that yields strong and robust performance for the identification of novel cell types, even with imbalanced datasets and for rarely occurring cell types. AnnoGCD is computationally efficient and only requires partially labeled data to correctly classify samples in known classes and automatically discover novel cell types.

## Related work

This paper addresses the problem of Generalized Category Discovery for automatic cell type annotation from single cell RNA-seq data. A summary of the problem setting of interest is given in Table 1, and more details follow.

**Table 1.** Comparison of different problem settings

Problem setting	Label data	Unlabeled data	
		Known classes	Unknown classes
Supervised learning	✓	✗	✗
Semi-supervised learning	✓	✓	✗
Robust Semi-supervised learning	✓	✓	Reject
Novel Class Discovery	✓	✗	✓
Generalized Category Discovery	✓	✓	✓

In the supervised learning setting, models are trained exclusively on labeled samples and they are only able to classify data (both labeled and unlabeled) into known classes. For semi-supervised and robust semi-supervised learning, the model leverages both labeled and unlabeled data, with the latter only classifying the known and rejecting the novel classes. Novel Class Discovery assumes that only novel classes exist in the unlabeled set and it is unable to re-discover known classes. Generalized Category Discovery aims to generalize Novel Class Discovery to further recognize the known classes in the unlabeled set.

### Semi-supervised learning

Semi-supervised learning (SSL) combines a small amount of labeled data with a large amount of unlabeled data during training. These hybrid approaches are particularly useful for settings in which labeling data is expensive or time-consuming (15). Traditional semi-supervised methods work under the assumption that the unlabeled set contains instances from the same classes as a given label set. As such, they cannot be trivially extended to discover novel classes. On the other hand, robust SSL methods (16) relax this assumption by assuming that instances from novel classes may appear in the unlabeled test set. However, robust SSL approaches aim to reject instances from novel classes, which are treated as out-of-distribution instances.

### Generalized Category Discovery

As proposed in (17), Novel Class Discovery aims to label the unknown samples with novel classes starting from a disjoint set of labeled examples. Since these approaches assume that only novel classes exist in the unlabeled set, they cannot identify the known classes which surely exist in an open-world setting. Introduced in (5), Generalized Category Discovery aims to generalize Novel Class Discovery to further recognize the known classes in the unlabeled set. Notable approaches include ORCA (18), a deep learning pipeline combining a supervised objective on the known classes and a pairwise objective on unlabeled data to generate pseudo-labels, and Opencon (19), which uses a contrastive loss to learn a compact representation space for both known and novel classes and a prototype-based out-of-distribution (OOD) detection to separate known and novel data.

### Cell type annotation

Automatic cell annotation is pivotal for research on disease progress and tumour microenvironments (20). In essence, computational methods aim at detecting a gene expression pattern in a cell or cluster that corresponds to the gene expression signature of a recognized cell type or state, leading to the assignment of a specific label. Several works proposed the use of machine learning methods for automatic cell type annotation. CaSTLe (21) employs an XGBoost classification model; CHETAH (21) requires a reference scRNA-seq dataset from which it constructs a hierarchical classification tree which is then traversed to classify the input cells; scPred (22) combines Principal Component Analysis (PCA) and a Support Vector Machine model for cell type classification; introduced in (23) and based on the original BERT (24), scBERT was recently proposed to infer cell types from single-cell RNA-seq data. All the aforementioned methods, while successful in the predictive task, suffer from two major drawbacks: (i) they require a large amount of labeled data for supervised training, and (ii) they are unable to infer cell types not seen during training.

## Problem statement

The objective of GCD entails classifying instances belonging to established known classes and identifying novel classes within unlabeled data. This task starts from a labeled dataset containing only the known classes (Figure 1). Consider the given labeled training data $\mathcal {X}^L = \lbrace x_i,y_i\rbrace$ with $y_i \in \mathcal {Y}^L$. All classes in $\mathcal {Y}^L$ are considered known and $K = \vert \mathcal {Y}^L \vert$ is the number of known classes. The unlabeled data $\mathcal {X}^U$ consists of samples of known classes from $\mathcal {Y}^L$ as well as instances of novel classes. Note that we do not have access to the labels in $\mathcal {X}^U$, nor do we know the number of novel classes. The known and novel class sets are constructed as non-intersecting sets, and we denote the underlying label set as $\mathcal {Y}^{all}$ where $\mathcal {Y}^L \subset \mathcal {Y}^{all}$. The learning task is to assign to all the unlabeled instances in $\mathcal {X}^U$ classes from $\mathcal {Y}^{all}$. While applicable to any kind of tabular data, in this paper we consider the problem of recognizing known cell types and inferring previously unseen cell types from unlabeled single-cell RNA sequencing data. Consequently, for the rest of the paper, we use the terms ‘class’ and ‘cell type’ interchangeably.

## Materials and methods

The proposed AnnoGCD framework consists of two main components: (i) a semi-supervised block and (ii) an unsupervised block. This two-step approach ensures that, during the first semi-supervised phase, which aims to assign to samples in $\mathcal {X}^U$ the classes in $\mathcal {Y}^L$, the unknown examples belonging to the known classes would be identified and assigned the correct labels. Having re-discovered the existing cell types in $\mathcal {X}^U$, the unsupervised block is then employed to identify the novel classes. We detail each block and its components in the next sections.

### Semi-supervised block

**Figure 2.**
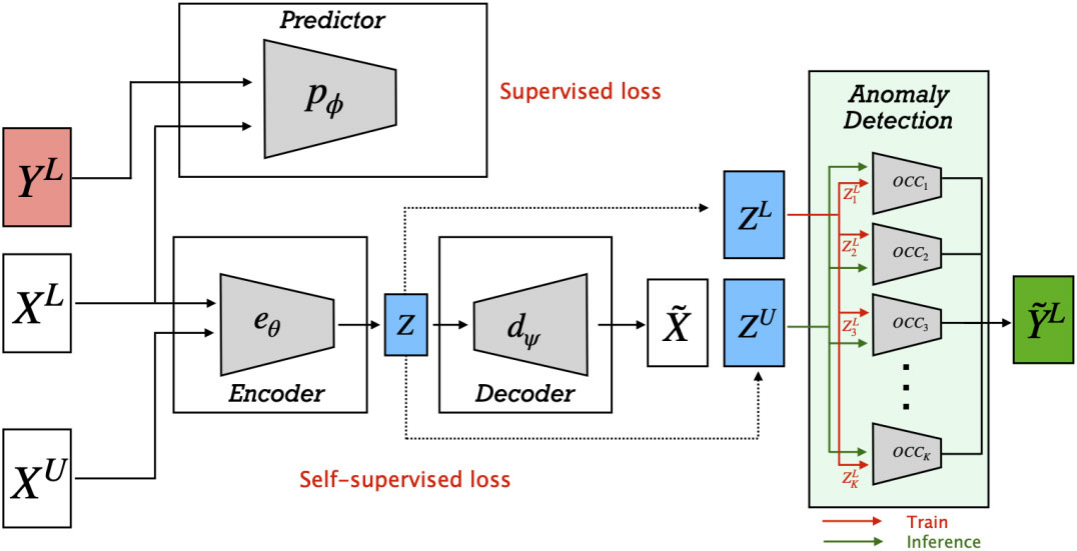
The semi-supervised block, which aims to re-discover samples belonging to the known *K* classes, is composed of a data encoder model (*e*_θ_), a data decoder model (*d*_ψ_) and a predictor model (*p*_ϕ_). The encoder maps the input data to a latent representation; the decoder reconstructs the initial data; a predictor learns to classify the known samples in the correct labeled classes. After the joint training of *e*_θ_, *d*_ψ_ and *p*_ϕ_, a collection of *K* One-Class Classifiers (OCCs) is trained using the latent embeddings to determine whether each of the unlabeled samples belongs to one of the known classes.

We start by training a semi-supervised block, shown in Figure 2, to classify the unknown samples in one of the known *K* classes. The semi-supervised block is composed of a data encoder model *e*_θ_, a data decoder model *d*_ψ_ and a predictor model *p*_ϕ_. The encoder is used to map the input data $\mathcal {X}$, both labeled and unlabeled, to a latent representation $\mathcal {Z}$ of fixed dimension: $e_{\theta } : \mathcal {X} \rightarrow \mathcal {Z}$. Any choice of encoder can be used depending on the input data, such as CNN for image data, Multi-layer Perceptron (MLP) for tabular data or Graph Neural Network (25) for relational data. A decoder is then used to reconstruct the initial data from $\mathcal {Z}$. Along with the encoder–decoder module, we train a predictor $p_{\phi } : \mathcal {X}^L \rightarrow \mathcal {Y}^L$ which learns to classify the known samples in the correct labeled classes. After the joint training of the encoder, decoder and predictor, given the presence of known cell types in the unlabeled samples, we use the latent representations $\mathcal {Z}^L$ of the labeled cells to train *K* OCCs, where *K* is the number of known classes. In detail, each one of the OCC classifiers—name it *k*-OCC—is trained using labeled samples for class *k* ($\mathcal {Z}^L_k$) to recognize instances of a known class *k*, where *k* ∈ [1, 2, …, *K*]. After training, the encoded representations of the unknown data $\mathcal {Z}^U$ are fed as input to all the *K* OCCs to perform inference and to determine whether an unlabeled sample belongs to one of the known *K* classes.

**Semi-supervised labelling via consensus:** The OCCs aim to detect whether an unknown sample was likely to have been generated by the underlying distribution of one of the known classes. Given an unknown sample *x*^*u*^, it is possible that (i) a single OCC predicts *x*^*u*^ to belong to its class, (ii) multiple OCCs predict *x*^*u*^ to belong to their classes or (iii) none of the OCCs predicts *x*^*u*^ to belong to its class. For the first case, we simply assign label *k* predicted by the *k*-OCC to sample *x*^*u*^. If *x*^*u*^ is predicted to potentially belong to multiple classes, we use a simple nearest neighbor approach, where the most common label amongst the top *N* neighbors of *x*^*u*^ from the label set is assigned as a label. Finally, if none of the OCCs predicts *x*^*u*^ to belong to its class, *x*^*u*^ remains unlabeled as it is probably significantly different from all the *K* known classes.

**Loss functions:** We train the semi-supervised block using two loss functions: (i) self-supervised mean-squared error (MSE) loss on the entire data and (ii) cross-entropy loss on the labeled data. In detail, using the notation introduced in the previous sections and in Figure 2, we define the self-supervised encoder-decoder loss on the entire dataset as:


(1) \begin{eqnarray*} \mathcal {L}_{\text{MSE}} = \frac{1}{M} \sum _{i=1}^{M} \Vert x_i - d_{\psi }(e_{\theta }({x}_i)) \Vert ^2 \end{eqnarray*}

and the loss on the labeled data as:


(2) \begin{eqnarray*} \mathcal {L}_{\text{label}} = -\frac{1}{M} \sum _{i=1}^{M} \sum _{j=1}^{K} y_{i,j}^l \cdot \log (\hat{y}_{i,j}^l) \end{eqnarray*}

where *M* is the total number of samples, $y_{i,j}^l$ is the true label indicator for a labeled sample $x_i^l$ and class *j*, *K* represents the number of classes, and $\hat{y}_{i,j}^l$ is the probability predicted by the predictor *p*_ϕ_ for $x_i^l$ belonging to class *j*.

The total loss is then given by:


(3) \begin{eqnarray*} \mathcal {L} = \alpha \mathcal {L}_{\text{MSE}} + \beta \mathcal {L}_{\text{label}} \end{eqnarray*}

where the hyper-parameters α and β are automatically learned during the joint training of the encoder, decoder and predictor, allowing the model to dynamically adjust their values for optimal performance.

### Unsupervised block

**Figure 3.**
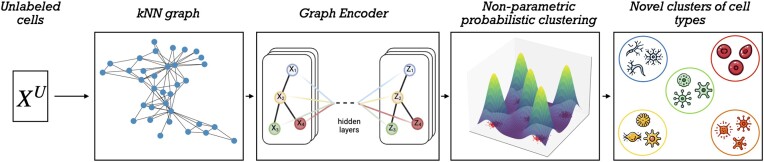
Starting from the remaining cells in $\mathcal {X}^U$, the unsupervised block constructs a k-Nearest Neighbors (kNN) graph, where edges are created between samples that are among the k-nearest neighbors of each other. To exploit the local neighborhoods and cell-to-cell similarities, the kNN graph is fed to a graph encoder model, Deep Graph Infomax (DGI) in our case, to learn node representations in an unsupervised manner. Using the latent representations learned from DGI, a Dirichlet Process Mixture Model is used to automatically infer the number of novel classes of cell types and perform clustering.

While the semi-supervised block serves to classify the unknown samples in $\mathcal {X}^U$ into the correct known cell type classes, it does not allow the discovery of novel classes. By integrating an unsupervised block in the AnnoGCD framework, shown in Figure 3, we aim to classify the samples which the semi-supervised block failed to label due to their significant difference from the known classes. The unsupervised block involves: (i) constructing a graph from the remaining unlabeled samples in $\mathcal {X}^U$, (ii) generating meaningful embeddings for each node of the graph based on neighbourhood information and (iii) identifying novel classes (and the number of novel classes) using a non-parametric probabilistic clustering approach.

**Graph construction:** While for the re-discovery of known classes, we could exploit the presence of the labeled examples through the predictor *p*_ϕ_, the remaining cells in $\mathcal {X}^U$ lack of any annotation. As such, we developed an unsupervised block exploiting the similarities of the unknown samples. In detail, from the unlabeled set in $\mathcal {X}^u$, we construct a graph *G* = (*V*, *E*) where each node *v* ∈ *V* is a cell, and interaction between two cells is described by an edge *e* ∈ *E*. Different ways exists to define the connectivity of *G* (for example, computing the correlation between the features of all pairs of nodes and creating an edge between two nodes if their correlation exceeds a certain threshold to control the sparsity of the graph). For our application, we considered a simple and efficient kNN graph, where edges are created between samples that are among the k-nearest neighbors of each other.

**Graph encoder:** To exploit the local neighborhoods and cell-to-cell similarities, we fed the constructed kNN graph as input to the Deep Graph Infomax (DGI) model to learn a lower-dimensional representation for each cell. Introduced in (26), DGI is an approach for learning node representations within graph-structured data in an unsupervised manner. In essence, DGI works by maximizing local mutual information by learning a node representation which captures the global information content of the entire graph. That is achieved through an encoder that maps the input feature and adjacency matrix to an embedding space. The encoder is composed of graph convolutional layers for aggregating features over neighbouring nodes with a parametric rectified linear unit as the activation function. As a proxy for maximizing the local mutual information, a discriminator is used to assign probability scores which evaluate how much the graph level information is contained in a local node representations. DGI is proposed to learn a representation of each sample that takes into account both the expression and the nearest neighbors connectivity structures.

**Non-parametric probabilistic clustering:** Bayesian nonparametric (BNP) techniques address model complexity in a fundamentally different way compared to conventional methods. While the number of parameters remains fixed in conventional analytics, BNP methods operate within a statistical framework characterized by the potential for an infinite number of parameters. One notable statistical framework is a stochastic process known as the Dirichlet Process (DP). Due to their flexibility in identifying clusters from data, Dirichlet Process Mixture Models (DPMMs) have become popular in a large number of applications (27,28). DPMMs are an extension of finite mixture models where the number of components (clusters) is not fixed a priori. Instead, they use a DP as a prior distribution over the mixture components, enabling models to automatically determine the appropriate number of clusters by analyzing the posterior mean or mode of the weight of each component. Having learned a latent representation for each node of *G* using DGI, we employ a DPMM to automatically infer the novel classes of cell types.

### Final label refinement via consensus

Once both the semi-supervised and unsupervised blocks of AnnoGCD are executed, all samples in $\mathcal {X}^U$ are assigned either to one of the known *K* classes or to a novel cell type. In the final step of the proposed pipeline, we perform a simple label refinement involving a nearest neighbor procedure, where all the cells are assigned the most common label among their top neighbors in $\mathcal {X}^U$.

## Datasets

To evaluate the proposed approach, we made use of five public multimodal datasets: the human bone marrow mononuclear cells (BM-CITE) (29), the human peripheral blood mononuclear cells from lung (LUNG-CITE) (30), the human peripheral blood mononuclear cells (PBMC-multiome) (accessible under: https://www.10xgenomics.com/), the human PBMC cells measured with the DOGMA-seq protocol (PBMC-DOGMA) (31), and the human PBMCs measured with the TEA-seq protocol (PBMC-TEA) (32). For our evaluation, we only focus on single-cell RNA data from these datasets. Every cell in each dataset is annotated with the corresponding cell type, and the pre-processing of the single-cell sequencing data followed that described in (33). For each dataset, we removed the cells whose annotated types appeared less than a pre-determined threshold in the entire dataset (less than 100 times in our case) and computed the Gini index as a measure of the degree of inequality in the distributions of the different cell types. For each dataset, we selected 50% of the classes to act as known classes and the remaining 50% as unknown classes; moreover, to allow for re-discovery in the unlabeled set, 70% of the known classes are labeled, with the unknown and the rest of the known all considered unlabeled. A summary of the datasets used for evaluation, the split between novel and known cell types and their imbalance (Gini) indexes are reported in Table 2.

**Table 2.** Datasets used for evaluation of the proposed framework

Dataset	Species	Protocol	Organ	No. of cells	Known types	Novel types	Gini Index
BM-CITE	Human	CITE-seq	Bone Marrow	30 672	12	11	0.89
LUNG-CITE	Human	CITE-seq	PBMC&Lung	10 470	8	8	0.85
PBMC-Multiome	Human	Multiome	PBMC	11 787	6	6	0.84
PBMC-TEA	Human	TEA-seq	PBMC	25 517	5	4	0.84
PBMC-DOGMA	Human	DOGMA-seq	PBMC	13 763	5	5	0.65

## Experimental results

**Evaluation metrics:** We evaluated the performance of AnnoGCD on both known and novel classes. For the re-discovery of known cell types, resulting from the semi-supervised block, we simply computed the classification accuracy between the generated labels and the ground truth labels. For the unknown classes, the clustering accuracy is calculated by solving the prediction-target class assignment based on the Hungarian algorithm. Proposed in (34), the Hungarian algorithm is a combinatorial optimization algorithm that solves the assignment problem in polynomial time, ensuring the optimal matching between predicted and true labels. Finally, we also evaluated the overall accuracy for both known and novel classes.

**Experimental details:** We applied Principal Component Analysis (PCA) to reduce the dimensionality of the single-cell RNA data, and selected the first 64 principal components as input to our pipeline. For the semi-supervised block, we employed a simple Multilayer Perceptron (MLP) as predictor *p*_ϕ_ (Figure 2) with two hidden layers of dimension 32, where every linear layer is followed by Relu non-linearity and softmax on the final layer. We also employed a simple three-layered autoencoder where each linear layer but the final one is followed by Relu to learn a hidden representation of size 32. After optimizing the loss in Equation 3 for 500 epochs, we trained *K* One-Class Support Vector Machine Classifiers (OC-SVMs) with Radial Basis Function kernels (35). The number *N* of nearest neighbors we used to generate a consensus for the semi-supervised labelling was 20, while 10 neighbors were employed for the final refinement. For the unsupervised block, we constructed a k-Nearest Neighbors (kNN) graph using the nearest 50 neighbors for each sample and trained a two-layered DGI model for 500 epochs to learn a lower dimensional representation of size 32 for each cell. Finally, we clustered the unknown samples using a Dirichlet Process Mixture Model (DPMM) to identify the novel classes. Specifically, we first fit a DPMM with a large number of components (20 in our case) and then used the posterior weight of each component to select the number of clusters. On all datasets, we used a fixed threshold *t* to identify the significant components; that is if the posterior weight π_*i*_ ≥ *t*, component *i* is considered significant. We empirically selected *t* = 0.05 for all our experiments. The above mentioned hyperparameters were chosen using cross validation only on the known classes (whose labels are available) as described in (36) to avoid data leakage. In detail, for each cross validation split, the instances of around half of the known classes are selected to form another set whose labels are hidden ($\mathcal {X}^{hid}$). After training the model with this new data split, we evaluated its performance on the instances of $\mathcal {X}^{hid}$ since their labels are available. To evaluate a given combination of hyperparameters, this approach is applied to all the splits, and performances on the hidden classes are averaged. The hyperparameter combination that achieved the best performance is selected and applied to the full dataset (36). Table 3 gives a comprehensive list of the evaluated hyperparameters and configurations.

**Table 3.** Evaluated hyperparameters for the experimental setup

Hyperparameter	Evaluated Values
Number of Principal Components	{32, **64**, 128}
MLP Hidden Layers	{**2**, 3}
MLP Hidden Layer Size	{16, **32**, 64}
Autoencoder Hidden Layers	{2, **3**}
Autoencoder Hidden Layer Size	{16, **32**, 64}
Masked autoencoder (37)	{Yes, **No**}
OC-SVM Kernel	{Linear, **Radial Basis Function**}
Number of Nearest Neighbors (*N*)	{10, **20**, 50}
Final Refinement Neighbors	{**10**, 20}
kNN Graph Neighbors	{20, **50**, 100}

The best hyperparameters, shown in bold, were chosen using cross validation on the known classes (whose labels are available) as described in (36).

**Baselines:** We start by comparing the proposed approach to two GCD pipelines: Openworld Semi-supervised Novel Class Discovery (OpenNCD) (38) and Projection-Based NCD (PBN) (36). OpenNCD leverages contrastive learning to create class prototypes that are progressively refined for classifying known and novel data. While effective in its domain and as with most GCD frameworks, OpenNCD is primarily designed for imaging data and relies heavily on feature extraction techniques, such as ResNet-18 (39), which are less suitable for non-vision data like single-cell RNA sequencing (scRNA-seq). Additionally, given the wide availability of image datasets, OpenNCD does not adequately handle imbalanced datasets, which are common in biological data. To the best of our knowledge, PBN is the only other method developed to tackle the problem of GCD in the tabular context. It consists of an encoder that learns a shared representation between the known and novel classes, a classification network trained on the latent representations to distinguish the known classes, and a decoder that reconstructs the data for both known and novel classes. Once the encoder, decoder and classification network have been trained, the unlabeled data is projected by the trained encoder into the latent space and then clustered to discover novel classes. While PBN relies on a simple clustering process after learning a shared latent space, AnnoGCD employs a two-step approach based a semi-supervised block for known class classification and an unsupervised block for novel class discovery, providing a more robust discovery mechanism, especially in scenarios characterized by strong class imbalance and high-dimensional data like scRNA-seq.

**Comparison with baselines:** To allow for a fair comparison, we replaced the ResNet-18 encoder and used the first 64 principal components as input to OpenNCD. We performed grid search to identify the optimal learning rate, temperature scale τ and batch size. Similarly for PBN, we performed grid search to identify the optimal number of layers for the encoder network, the best learning rate, dropout rate and latent space dimension for each scRNA dataset independently. The full list of searched hyperparameters for the baseline methods is given in Table 4. Furthermore, for both OpenNCD and PBN, the number of novel classes to discover was given as an additional input. Table 5 shows the accuracy comparison of our AnnoGCD and the two baselines for known, novel, and all classes over three different iterations. Our model exhibits the best performances in re-discovering known classes for four out of five datasets. AnnoGCD was also able to identify the novel classes with the highest accuracy in all but one dataset. When compared in terms of accuracy over all classes, our pipeline reached the best results across all datasets. As shown in Table 5, AnnoGCD was successful in the task of identifying known and novel cell types even in biological datasets characterized by strong label imbalance, as indicated by the Gini indexes in Table 2.

**Table 4.** Grid searched parameters for the baselines

Method	Parameter	Values
OpenNCD (38)	Learning Rates	{0.0001, 0.001, 0.01}
	Taus (τ)	{0.1, 0.3, 0.5}
	Batch Size	{128, 256, 512}
PBN (36)	Encoder Layers	{2, 3}
	Learning Rates	{0.0001, 0.001, 0.01}
	Dropout Rates	{0.2, 0.4, 0.6}
	Latent Space Sizes	{16, 32, 64}

**Table 5.** Comparisons for known, novel and all classes over three different iterations

Dataset	Known			Novel			All		
	Ours	(38)	(36)	Ours	(38)	(36)	Ours	(38)	(36)
BM-CITE	**0.82** ± 0.004	0.69 ± 0.015	0.64 ± 0.010	0.67 ± 0.046	**0.74** ± 0.012	0.48 ± 0.009	**0.79** ± 0.031	0.74 ± 0.018	0.54 ± 0.011
LUNG-CITE	**0.89** ± 0.002	0.54 ± 0.010	0.42 ± 0.008	**0.74** ± 0.004	0.73 ± 0.014	0.49 ± 0.006	**0.87** ± 0.001	0.72 ± 0.016	0.46 ± 0.010
PBMC-Multiome	**0.95** ± 0.004	0.78 ± 0.010	0.34 ± 0.009	**0.76** ± 0.053	0.68 ± 0.012	0.69 ± 0.011	**0.83** ± 0.028	0.72 ± 0.012	0.48 ± 0.010
PBMC-TEA	0.68 ± 0.008	**0.73** ± 0.016	0.71 ± 0.010	**0.72** ± 0.024	0.64 ± 0.013	0.32 ± 0.009	**0.81** ± 0.015	0.73 ± 0.017	0.38 ± 0.010
PBMC-DOGMA	**0.90** ± 0.015	0.72 ± 0.013	0.37 ± 0.010	**0.50** ± 0.040	0.47 ± 0.014	0.44 ± 0.012	**0.76** ± 0.038	0.63 ± 0.016	0.39 ± 0.010

For each dataset, 50% of the classes are regarded as known and the rest 50% as unknown. In addition, 70% of the known classes are labeled, with the unknown and the rest of the known all considered unlabeled. Best results are shown in **bold**.

**Ablation studies:** To investigate the influence of the supervised component in the semi-supervised block, we trained the proposed pipeline without the predictor *p*_ϕ_ (Figure 2). The accuracy of the semi-supervised block, which measures the ability to re-discover existing classes, is reported in the first column of Table 6. Comparing the results without the predictor *p*_ϕ_ from Table 6 to the accuracy on the known classes in Table 5, it is clear that the presence of the supervised component in the semi-supervised block is useful to slightly increase the prediction performances. Similarly for the unsupervised block, we evaluated its accuracy to discover novel classes when discarding the locality information provided by the graph embeddings. In this case, the principal components were used to perform novelty detection by the non-parametric probabilistic clustering. Comparing the results in the second column of Table 6 to the accuracy on the novel classes in Table 5 clearly shows that integrating the locality and connectivity structure by means of graph embeddings is crucial for correctly identifying novel classes of cell types.

**Table 6.** (*left*) Accuracy of the semi-supervised block without the supervised loss. (*right*) Accuracy of the unsupervised block without the graph embeddings. Means and standard davations are computed over three different iterations

Dataset	w/o Predictor	w/o Graph Embeddings
BM-CITE	0.81 ± 0.002	0.48 ± 0.014
LUNG-CITE	0.88 ± 0.005	0.44 ± 0.008
PBMC-Multiome	0.94 ± 0.003	0.55 ± 0.071
PBMC-TEA	0.68 ± 0.007	0.69 ± 0.009
PBMC-DOGMA	0.87 ± 0.010	0.53 ± 0.041

**Figure 4.**
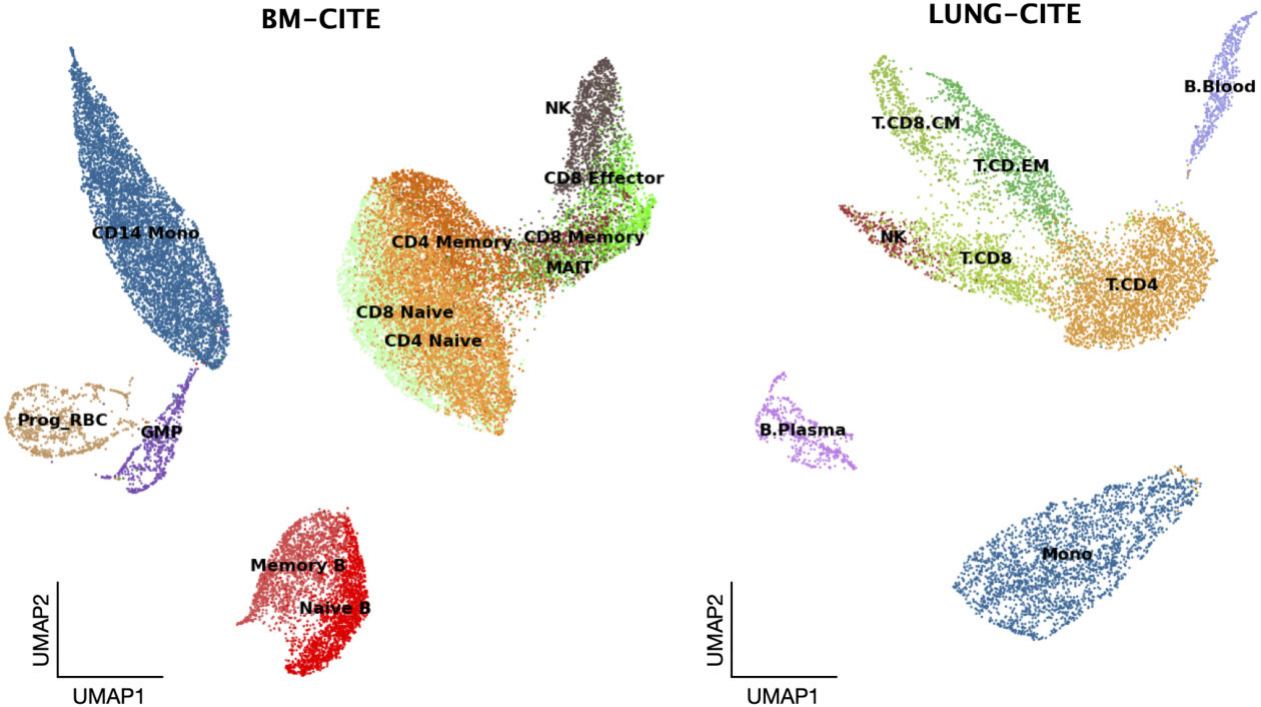
Qualitative visualization of the cell embeddings for the BM-CITE dataset (*left*) and LUNG-CITE (*right*) dataset. These visualizations demonstrate well-defined clusters, indicating AnnoGCD’s proficiency in learning meaningful biological representations of cell types.

**Embeddings visualization:** Figure 4 shows the UMAP projections of the embeddings $\mathcal {Z}$ learned by the semi-supervised block for two of the evaluated datasets: BM-CITE (left) and LUNG-CITE (right). In the left plot of Figure 4, we observe well-separated clusters corresponding to different cell types, indicating that the semi-supervised block effectively captures the intrinsic structure of the data and separates distinct cell types. The clusters are annotated with cell type labels predicted by the OCCs, such as CD14 Monocytes (CD14 Mono), CD8 Naive, CD4 Naive, CD4 Memory, CD8 Memory, CD8 Effector, NK (Natural Killer) cells, MAIT (Mucosal-associated invariant T cells), Memory B, Naive B, Prog RBC (Progenitor Red Blood Cells) and GMP (Granulocyte-Monocyte Progenitors). The clear separation between these clusters suggests that the model accurately distinguishes between different known cell types based on their specific gene expression profiles. The right plot of Figure 4 shows labeled clusters for the LUNG-CITE dataset. The labels include B Plasma cells, CD8 CM (Central Memory CD8 T cells), CD4 EM (Effector Memory CD4 T cells), NK (Natural Killer) cells, CD8 (T cells), CD4 (T cells), Monocytes and B Blood cells. Similarly to the left plot, this visualization demonstrates well-defined clusters, indicating the model’s proficiency in learning meaningful representations of cell types. The clustering of T and B cells into distinct groups showcases AnnoGCD’s ability to recognize and categorize different immune cell subtypes accurately. Finally, by automatically learning representations able to form biologically relevant clusters, AnnoGCD shows potential utility in enhancing the understanding of cellular heterogeneity in various biological contexts.

**Quantity of labeled samples:** Table 5 shows AnnoGCD accuracies for each dataset when 50% of the classes are regarded as known and the remaining 50% as unknown. In addition, 70% of the known classes were labeled, with the unknown and the rest of the known all considered unlabeled. We investigated the ability of our approach to identify known and novel cell types, with varying numbers of labeled samples. Results of this evaluation for one of the datasets, BM-CITE, are reported in Table 7. While the accuracy of the semi-supervised block remains high, since most of the re-discovered cell types are correctly classified, decreasing the number of labeled samples results in a larger proportion of known examples being missed by the semi-supervised block. As a result, the identification of new classes of cell types is hindered and the performances decrease as more and more samples are being removed from the label set, as is clearly evident from Table 7.

**Table 7.** Accuracy for different numbers of labeled samples on the BM-CITE dataset

Dataset	Labeled samples	Known	Novel
BM-CITE	70%	0.82	0.70
	50%	0.82	0.65
	30%	0.82	0.58

**Mouse model organism:** Since ORCA (18) is a popular deep learning pipeline for open-world semi-supervised learning, and it was applied in the original manuscript to a single-cell Mouse Ageing Cell Atlas (40), we use it as a further comparison to our pipeline. The Mouse Ageing Cell Atlas consists of 93 718 cells from 50 cell types collected across 23 organs of the mouse model organism. Similarly to ORCA, we selected the 2866 most highly variable genes and used 50% of the classes as seen and 50% as novel classes. We then selected 50% of seen classes as the labeled dataset, and the rest as the unlabeled set. The largest class has 13 268 examples, while the smallest class has 479 examples. Given the larger dataset, we trained our semi-supervised block with a larger hidden dimension of size 64, and optimized the loss in Equation (3) for 1000 epochs.

Results of this comparison are shown in Table 8. In terms of re-discovering known classes, our approach was able to outperform ORCA, which showed better results in identifying novel classes of cell types. Overall, our approach showed higher accuracy on the entire dataset, outperforming ORCA by a large margin. While advantageous for reducing bias towards seen classes and improving novel class discovery, the uncertainty adaptive margin mechanism that ORCA employs slows down the learning of known classes. In contrast, AnnoGCD’s semi-supervised learning block effectively leverages labeled data to learn known classes with higher precision and ensures stronger classification accuracy for known categories.

**Table 8.** Comparison with ORCA (18) on the single-cell Mouse Ageing Cell Atlas (40)

Method	Known	Novel	All
Ours	**0.92**	0.60	**0.89**
ORCA (18)	0.89	**0.65**	0.73

Best results are shown in **bold**.

**Over-representation analysis of novel clusters:** Over-representation analysis (ORA) is a widely used statistical method for interpreting gene expression data to identify cell types. It involves comparing the proportion of a set of genes of interest (those that are differentially expressed) that are associated with a particular biological pathway or function, against the proportion expected by chance in a reference set of genes. ORA is particularly valuable in scRNA-seq sequencing studies, where it helps in characterizing and identifying cell types based on their specific gene expression profiles (41). To identify the functional enrichment of the novel clusters identified by AnnoGCD, we performed ORA using decoupleR (42). In detail, to annotate single cell clusters, we used cell type specific marker genes reported in PanglaoDB (43) to predict the most likely cell types per cluster. Using this approach on the BM-CITE dataset, we were able to map most of the cell types identified amongst the novel clusters to their ground truth annotations. Results are reported in Table 9. Out of the 11 unknown cell types (Table 2), we were able to automatically map 6 of the cell types predicted by decoupleR to their annotated type as shown in Table 9. The remaining unmapped clusters were manually annotated by inspecting their over-represented genes. For instance, cluster 14 exhibited over-expression of MKI67, TOP2A, STMN1 and TYMS genes, known to be cell cycle and proliferation markers (44) and were matched to Hematopoietic Stem Cells (HSC) in the reference dataset. Cluster 15 showed high expression of hemoglobin beta (HBB) and ribosomal proteins (RPL3, RPS4X) which is indicative of erythroid lineage (45). Since they are closely related to Megakaryocyte Progenitors, they were matched to this cell type in the reference dataset. Using this approach, and combining our automatic pipeline with limited manual inspection, AnnoGCD was able to correctly identify cell type annotations for the unknown clusters of cells.

**Table 9.** Identification of cell types for the novel classes

Cluster	decoupleR (42)	Reference cell type
12	Gamma-delta T cells	Gamma-delta T cells (gdT)
13	Dendritic Cells	Conventional Dendritic Cells 2 (cDC2)
16	Monocytes/Macrophages	CD16 Monocytes (CD16 Mono)
17	Plasmacytoid Dendritic Cells	Plasmacytoid Dendritic Cells (pDC)
19	Plasma Cells	Plasmablasts
21	B cells	B cell progenitor

We performed ORA and used decoupleR (42) to identify the most likely cell type for each unknown cluster. In the last column, we show which cell type was matched in the reference dataset based on the ground truth annotations.

## Conclusion

In this study, we introduced a novel computational framework, named AnnoGCD, that effectively combines semi-supervised learning with unsupervised methods to tackle the limitations of traditional supervised settings in open-world scenarios, particularly for automatic cell type annotation from single-cell RNA sequencing (scRNA-seq) data. AnnoGCD builds on Generalized Category Discovery (GCD) and Anomaly Detection (AD) to address the challenges posed by the need for extensive labeled datasets and the presence of novel cell types in biological research. Our framework is designed to handle the realistic constraints of limited labeled data, budget limitations, and incomplete information, which are common in practical applications. In detail, AnnoGCD uses a semi-supervised block to classify known cell types, followed by an unsupervised block designed to identify and cluster novel cell types. The efficacy of AnnoGCD was validated using five public scRNA-seq datasets, showing superior performance in both known and novel cell type identification compared to existing methods. The proposed pipeline demonstrated robustness in datasets characterized by significant class imbalance, a common issue in real-world biological data. This robustness is critical for ensuring the reliable identification of rare or underrepresented cell types, which are often pivotal in understanding disease mechanisms and therapeutic targets. We showed that AnnoGCD was able to learn meaningful representations of cell types and that it allowed to automatically decipher cell type annotations for unknown clusters of cells. Beyond its application to single-cell RNA sequencing data, AnnoGCD holds significant potential for use in other domains that require the classification of known categories and the discovery of novel ones from complex, high-dimensional data and future work will involve assessing its performance across other types of tabular or structured data.

## Data Availability

Our code and the datasets used for evaluations are publicly available on GitHub: https://github.com/cecca46/AnnoGCD/. We also make them available on FigShare: https://doi.org/10.6084/m9.figshare.26889349.v1.
